# Inhibition SIRT1 to regulate FOXP3 or RORγt can restore the balance of Treg/Th17 axis in ulcerative colitis and enhance the anti-inflammatory effect of moxibustion

**DOI:** 10.3389/fimmu.2024.1525469

**Published:** 2025-01-10

**Authors:** Yuanbing Zhu, Yuemei Wang, Xiaotong Zuo, Shuqing Liu, Lishuang Cao, Junmeng Wang, Qingqing Yang, Qianhui Huang, Qin Huang, Muqiu Tian, Yanling Ping, Qiaofeng Wu

**Affiliations:** ^1^ Acupuncture and Moxibustion College, Chengdu University of Traditional Chinese Medicine, Chengdu, Sichuan, China; ^2^ Acupuncture & Chronobiology Key Laboratory of Sichuan Province (Chengdu University of Traditional Chinese Medicine), Chengdu, Sichuan, China; ^3^ Key Laboratory of Acupuncture for Senile Disease (Chengdu University of Traditional Chinese Medicine), Ministry of Education, Chengdu, Sichuan, China; ^4^ Institute of Acupuncture and Homeostasis Regulation, Chengdu University of Traditional Chinese Medicine, Chengdu, Sichuan, China

**Keywords:** ulcerative colitis, acetylation, SIRT1, moxibustion, Treg/Th17, intestinal barrier

## Abstract

**Introduction:**

Ulcerative colitis (UC) is a chronic inflammatory disease. Patients with UC typically exhibit disruption of the Treg/Th17 immune axis, but its exact mechanism is still unclear.

**Methods:**

This study first analyzed RNA- seq data from public databases of humans and mice, and *in vitro* cytology experiments were conducted to induce or inhibit the expression of SIRT1. *In vivo*, UC mice were treated with moxibustion and SIRT1 inhibitor EX-527 to confirm the changes in the transcription factors identified through analysis of the datasets.

**Results:**

The results show that Treg/Th17 axis disruption is an important feature of UC. Differential gene expression and immune infiltration analysis showed that upstream transcription factors, including Forkhead box P3 (FOXP3), were significantly disrupted. *In vitro* cytology experiments, the results indicate that SIRT1 is activated in LPS induced inflammation, subsequently perturbing the Treg/Th17 immune balance axis. Finally, *in vivo* studies, the results have shown that administering EX-527 to inhibit SIRT1 leads to an increasing in FOXP3 expression and a decreasing in RORγt expression in UC colon tissue. In addition, the results indicate that traditional Chinese moxibustion can down regulate the expression of SIRT1, directly affecting the balance of Th17/Treg axis, and the combined use of EX-527 further improves the therapeutic effect of moxibustion.

**Conclusion:**

Our research shows that inhibition SIRT1 can regulate Treg and Th17 immune balance axis. This finding indicates a new important potential target for the treatment of UC.

## Introduction

Ulcerative colitis (UC)is a chronic inflammatory disease that affects the colon and rectum, characterized by recurrent mucosal inflammation. This leads to symptoms including abdominal pain, diarrhea, hematochezia, and other gastrointestinal issues (1), significantly impacting patients’ quality of life and posing substantial socioeconomic challenges (2). Despite the unclear etiology, dysregulated immune responses, particularly disruptions in the Treg/Th17 axis, are implicated in UC’s pathogenesis (3, 4). Therapeutically, restoring immune homeostasis by modulating the Th17/Treg balance is considered promising. Regulatory T cells (Tregs) are known to produce anti-inflammatory factors and immunosuppressive molecules, which inhibit the activity of various immune cells and suppress excessive immune response, maintaining immune homeostasis (5). Contrarily, T helper type 17 (Th17) is a major inducer of autoimmunity, driving inflammation through the production of various inflammatory cytokines. However, the precise upstream mechanisms underlying the restoration of the Th17/Treg balance remain elusive.

T cell stability is illuminated through the analysis of transcriptional profiles arising from the interplay of competing transcription factor gradients. FOXP3, a forkhead-winged-helix family member, is vital for Treg specification and function, being constitutively expressed in these cells. Conversely, Th17 cell differentiation is governed by retinoic acid receptor-related orphan receptor gamma t (RORγt), an isoform of *RORc*. Th17 cells and Treg cells, derived from a common naive CD4 T cell precursor, contributing to the heterogeneity of T cells. Post-translational modifications of transcription factors are considered a common target for exogenous intervention measures. The balance of FOXP and RORγt can determine the direction of initial T cell differentiation towards Treg or Th17 lineages, and is crucial for maintaining functional homeostasis in multiple organs such as the intestine (6). Acetylation modification is one of the most fundamental and critical regulatory mechanisms for FOXP3 and ROR γ t (7, 8). Therefore, investigating acetylation modifications at the transcriptional level may provide valuable insights into the Treg/Th17 axis mechanism and identify potential therapeutic targets.

In this study, we firstly conducted a comprehensive analysis of transcriptome data from both UC patients and UC mice to confirm the alteration of Tregs and Th17 protein acetylation levels, besides, silencing information regulator 2 related enzyme 1(sirtuin1, SIRT1), an NAD-dependent deacetylase, has been demonstrated to mediate various physiological processes, including oxidative stress, DNA damage and repair, metabolism, cell proliferation, and apoptosis (9). Evidence once reported SIRT1 is the main modifying enzyme that mediates the differentiation of naive CD4+T cells. Tregs cells and Th17 cells are important subset of CD4+T cells, FOXP3 and RORγt are core transcription factors for Tregs cells and Th17 cells respectively, the expression of FOXP3 and RORγt can affect the differentiation of naive T cells. Thus, we hypothesis that colonic SIRT1 can rebalance the Treg/Th17 axis by mediating the expression of core transcription factor, including FOXP3for Tregs and RORγt for Th17 cells. Next, we carried out *in vitro* cell experiments to explore whether SIRT1 can influence Treg/Th17 axis through the acetylation level of FOXP3 and RORγt. Finally, as we previously reported that moxibustion is a potential effective therapy method for UC (10), we used EX-527 to inhibit SIRT1 when given moxibustion, aiming to observe the effect of moxibustion combine with SIRT1 intervention. We hope to elucidate the mechanisms of UC from the modification of Treg/Th17 axis as well as to provide a new clue for a new effective treatment combination of traditional Chinese medicine and drugs for UC.

## Materials and methods

### Acquisition and analysis of RNA-seq data

Datasets of GSE227407 (including 50 UC tissues & 21 normal tissues) and GSE87466 (3 UC tissues & 3 normal tissues) from Gene Expression Omnibus (GEO) database were selected for analysis (11, 12). Quality control of the transcriptomics data and subsequent analysis were conducted as previously described (12). Differential expression analysis used by GEO2R. To perform the GSEA enrichment analysis, the software Clusterprofiler was employed, utilizing gene sets derived from Gene Ontology (GO). the CIBERSORT and the immuCellAI database (http://bioinfo.life.hust.edu.cn/web/ImmuCellAI) was employed to assess the infiltration of immune cells in the normal and UC group.

The RNA-seq datasets GSE214695 in this study were sourced from GEO (including 6 UC tissues and 6 normal tissues) (13), (http://www.ncbi.nlm.nih.gov/geo/). The Seurat package (version 4.0) was employed for the comprehensive processing, stringent quality control of the acquired data, as well as for conducting the differential expression analysis. The software EdgeR was utilized, using a fold change threshold of 1.5 and a significance level of Padj < 0.05. The scores of acetylation gene-set enrichment analysis were calculated by UCell (14).

The basic characteristics of the GSE dataset provided with **Table 1**.

**Table 1:** Basic characteristics of three UC datasets.

Datasets	RNA type	Platform	Experiment type	Sample size	Sample source	Organism	Year
GSE214695	RNA-seq	GPL18573	Expression profiling by high throughput sequencing	6 normal& 6 UC & 6 CD	Colonic mucosal	Homo sapiens	2023
GSE87466	mRNA	GPL13158	Expression profiling by array	21 normal& 87 UC	Colonic mucosal	Homo sapiens	2018
GSE227407	RNA-seq	GPL24973	Expression profiling by high throughput sequencing	3 normal & 3 UC& 3 EAST36	Colon tissue	Mus musculus	2023

### Mice

C57BL/6J mice (weight 26 ± 1 g, age 8-10 weeks) were supplied by SJA Laboratory Co. Ltd. (Hunan, China) and housed in a setting with a controlled temperature (20°C ± 2°C) with free accessing to diet and drinking water. All animal studies were conducted in line with the guidelines outlined in the Guide for the Care and Use of Laboratory Animals. The Institutional Animal Care and Use Committee (IACUC) at Chengdu University of Traditional Chinese Medicine (No.2024004) approved all experimental procedures.

### UC model inducing, moxibustion and EX-527 intervention

A 2.5% (w/v) DSS (43 kDa, MP Biomedicals) solution was used to provoke experimental colitis in mice for seven days. On the fifth day after DSS induction, the acupoints “Guan Yuan” (CV4) and “Zu San Li” (ST36) were chosen for the moxibustion. Specifically, throughout the treatment process, mice were secured in a customized apparatus that ensured full exposure of the “ST36”and “CV4” acupoints, the positioning of acupoints was based on the “Government Channel and Points Standard GB12346-90 of China” and “The Veterinary Acupuncture of China”, the specific acupoints location are as follows: The “ST36” is located laterally and posterior to the knee joint, approximately 2 mm below the fibular head. The “CV4” acupoint is situated in the lower abdomen, directly below the umbilicus by about 2 mm. During the moxibustion therapy, the moxa stick was consistently kept at a distance of 1.2-1.5 cm from the acupoints. Additionally, to control the temperature of the moxa and prevent skin burns, a thermometer was used throughout the procedure to maintain a constant temperature of 38°C ± 1°C. (**Supplementary Figure S4**). The control and DSS group mice were simply restrained without additional manipulation. Mice received daily moxibustion treatments lasting 15 minutes for a period of five days. In addition, SIRT1 inhibitor group received intraperitoneal injections of EX-527 (Abmole, USA) and administered at a dosage of 10 mg/kg daily for five successive days.

### Assessment of disease activity index

The daily body weight, fecal viscosity, and fecal occult blood changes in mice were recorded and assessed in terms of the disease activity index (DAI). Briefly, DAI was summarized by scoring these parameters as follows (15): (1) weight loss (0, no loss. 1, 1%-5% loss. 2, 6%-10% loss. 3, 11%-15% loss. and 4, over 15% loss), (2) stool consistency (0, normal. 2, loose stools. 4, watery diarrhea), (3) stool occult blood (0, no blood. 2, slight blood. and 4, gross blood). The DAI score was determined with the following equation: DAI = (weight loss score + stool consistency score + stool occult blood score)/3.

### Sample collection

After five days of moxibustion or EX-527 intervention, the experimental mice were euthanized under anesthesia, Blood samples were extracted from the eyeball. The length of the distal colon of the mice, measured from 1 cm above the anus to the rectum, was recorded. The distal colon tissues were processed by fixation with 4% paraformaldehyde, followed by embedding in paraffin and subsequent sectioning, or frozen at -80°C for further analysis.

### Histological observation

HE staining was performed for histological observation. Morphological changes in the distal colon of all mouse group were observed under an optical microscope. We further used electron microscopy to characterize the ultrastructure of the distal colonic mucosal epithelium.

### Immunofluorescence staining for confocal microscopy

FOXP3, ROR**γ**t, SIRT1, ZO-1 and Occludin were detected by confocal microscopy (TCS SP8, Leica, Germany). Paraffin sections were dewaxed, antigen repaired, incubated with bovine serum albumin (BSA), subsequently, the tissues were incubated overnight with 4°C primary antibodies specific to the proteins mentioned below: FOXP3,RORγt (Abcam, USA, used at 1: 200 dilution), SIRT1 (Proteintech, USA, used at 1: 200 dilution), Occludin (Proteintech, USA, used at 1: 200 dilution), ZO-1 (Proteintech, USA, used at 1:200 dilution). After washing, the slices were subjected to a 2-hour incubation at 37°C with the secondary antibody (Alexa Fluor 488, cy3, Bioss, China, diluted at 1:400 dilution). Five random areas were examined under the confocal laser scanning microscopy at 400× or 200× magnification.

### Western blot analyses

After extracting the proteins from the distal colon, the total protein content was separated by sodium dodecyl sulfate polyacrylamide gel electrophoresis (SDS-PAGE), following this, the proteins were transferred to PVDF. Blocking of the membranes was carried out using 5% non-fat dry milk in Tris-buffered saline for a 2-hour period at room temperature. Primary antibodies against SIRT1(Proteintech, used at 1:3000 dilution) and β-tubulin (Proteintech, used at 1:5000 dilution) were incubated with the membranes at 4°C overnight. Subsequently, the membranes were incubated for 2 hours at room temperature with secondary antibodies conjugated to HRP. Protein bands were revealed by ECL developing solution. Finally, ImageJ software was used for analysis.

### Fluorescein isothiocyanate–dextran permeability assay

The fluorescein isothiocyanate (FITC)-dextran permeability assay is commonly used to test the integrity of the intestinal barrier (Cani et al.,2008). The experimental procedures followed the established protocol. (Seifi et al.,2018). Before the experiment, mice had to be fasted for 4 hours. Subsequently, FITC-dextran (FD4; Sigma Aldrich) was administered to mice by gavage at a concentration of 600 mg/kg. Two hours later, blood was collected by extracting the eyeball and centrifuged at 4000 rpm for a duration of 10 min at 4°C. The plasma samples were protected from light and refrigerated at -80°C. FITC-dextran was diluted in phosphate-buffered saline to obtain standard solutions with concentrations ranging from 0.2 to 25 μg/mL, and 100 μL of diluted plasma samples, standard solutions, and blanks were transferred to black 96-well microplates. A fluorescence spectrophotometer (TECAN, Infinite M200) was used for the analysis, utilizing an excitation at 485 nm and emission at 528 nm. Finally, for each plasma sample, the FITC-dextran concentration was determined based on the standard curve.

### Flow cytometry

Spleen tissues were mashed and strained through a cell filter to obtain a single-cell suspension. Erythrocyte lysis buffer was employed to eliminate red blood cells from the spleen and blood. The suspension was then incubated with FITC-conjugated CD25 (eBioscience, USA), FITC-conjugated CD4 (Southern Biotech), PE-conjugated FOXP3 (BD Biosciences, USA), and PE-conjugated IL-17A (BD Biosciences, USA) at 4°C for 20 minutes. To label intracellular antigens, fixation and permeabilization steps were performed to allow cytoplasmic antibodies to access the interior of the cells. Finally, the enumeration of CD4+IL-17A+ or CD25+Foxp3+ lymphocytes in blood, spleen, and primary CD4+ cells was conducted using the ZE5 (Bio-rad, USA) flow cytometer.

### qRT-PCR

Total RNA was extracted from colon tissues with a total RNA extraction kit (TaKaRa,Kyoto, Japan). The concentration of RNA was measured, and complementary DNA (cDNA) was generated using a reverse transcription kit (TaKaRa, Shiga, Japan). The PCR setup was 20 μL, initiating with a denaturation step at 95°C for 5 minutes, followed by 40 cycles of denaturation at 95°C for 10 seconds and primer annealing at 60°C for 30 seconds. A melting curve was produced. The comparative expression levels of Foxp3 and RORγt mRNA in colon tissues were assessed using the 2-ΔΔCt method. The list of primer sequences is provided with **Table 2**.

**Table 2:** Primers sequences.

Name	Sequence(5’ to 3’)
*Foxp3*	Forward: TCCCTCCACTCCACCTAAA Reverse: CCTAATGCCTCCCAGAGC
*Rorγt*	Forward: GAACTTGGGGAACCAGAAC Reverse: TGGCATGTCTCTCGGAA
*β-actin*	Forward: GGCTGTATTCCCCTCCATCG Reverse: CCAGTTGGTAACAATGCCATGT

### Cell cultures

Colonic epithelial cells were extracted from the distal colon. The tissue was cut into small pieces and incubated with 1% type I and IV collagenase (Gibco, USA) for 20 minutes. Dulbecco’s Modified Eagle Medium/High glucose (DMEM) supplemented with 10% (v/v) fetal bovine serum (Gibco, USA) was added, and the cells were cultured in an environment consisting of 95% O_2_ and 5% CO_2_ in an incubator (Sanyo, Japan). For the subsequent experiments, the cells were passaged two or three times and seeded at a density of approximately 2 × 10^4^ cells per well in 24-well plates and cultivated for a total of 24 hours. lipopolysaccharide (LPS) (Solarbio, China), EX-527 were added in plates and incubated for 24 hours then supernatant was collected, and the cells were fixed with 4% paraformaldehyde for subsequent experimental assessments. CD4+T cells were purchased from MeisenCTCC and cultured in 24-well plates with RPMI 1640 medium (Fisher Scientific) which containing 10% FBS and respectively co-culture with10 µM EX-527 and SRT1720 HCL(Selleck, USA)for 72 h and 24 hours, then supernatant was collected for further measurement.

### Cytokine measurement

For *in vivo* serum collection, blood was obtained at indicated time points and allowed to clot for 40 minutes at room temperature. Subsequently, the samples were centrifuged at 2000g for 10 minutes at a 4°C environment. For *in vitro* experiments, the supernatants were centrifuged at 800g for 5 minutes then frozen at -80°C for further analysis. The levels of IL-10, TGF-β1, and TNF-α in the supernatant or serum were quantified using ELISA kits (MultiSciences, China), adhering to the provided instructions. The cytokine concentrations for each group were assessed based on the measured absorbance.

### Statistical analysis

All analyses were conducted utilizing GraphPad Prism Software (GraphPad Software, La Jolla, CA, USA). All data in the figures are expressed as means ± SD. Statistical evaluations were conducted utilizing either one-way or two-way ANOVA, followed by multiple pairwise comparisons employing Tukey’s or Bonferroni’s *post hoc* tests for multiple hypothesis testing. Student’s t tests were used for comparison between two groups. The Kolmogorov-Smirnov method was used to assess the normal distribution and equal variances of the data, with p-values below 0.05, 0.01, and 0.001 deemed statistically significant.

## Results

### Disruption of the Treg/Th17 axis in UC patient colon is characterized by the altered core transcription factors

**Figure 1:**
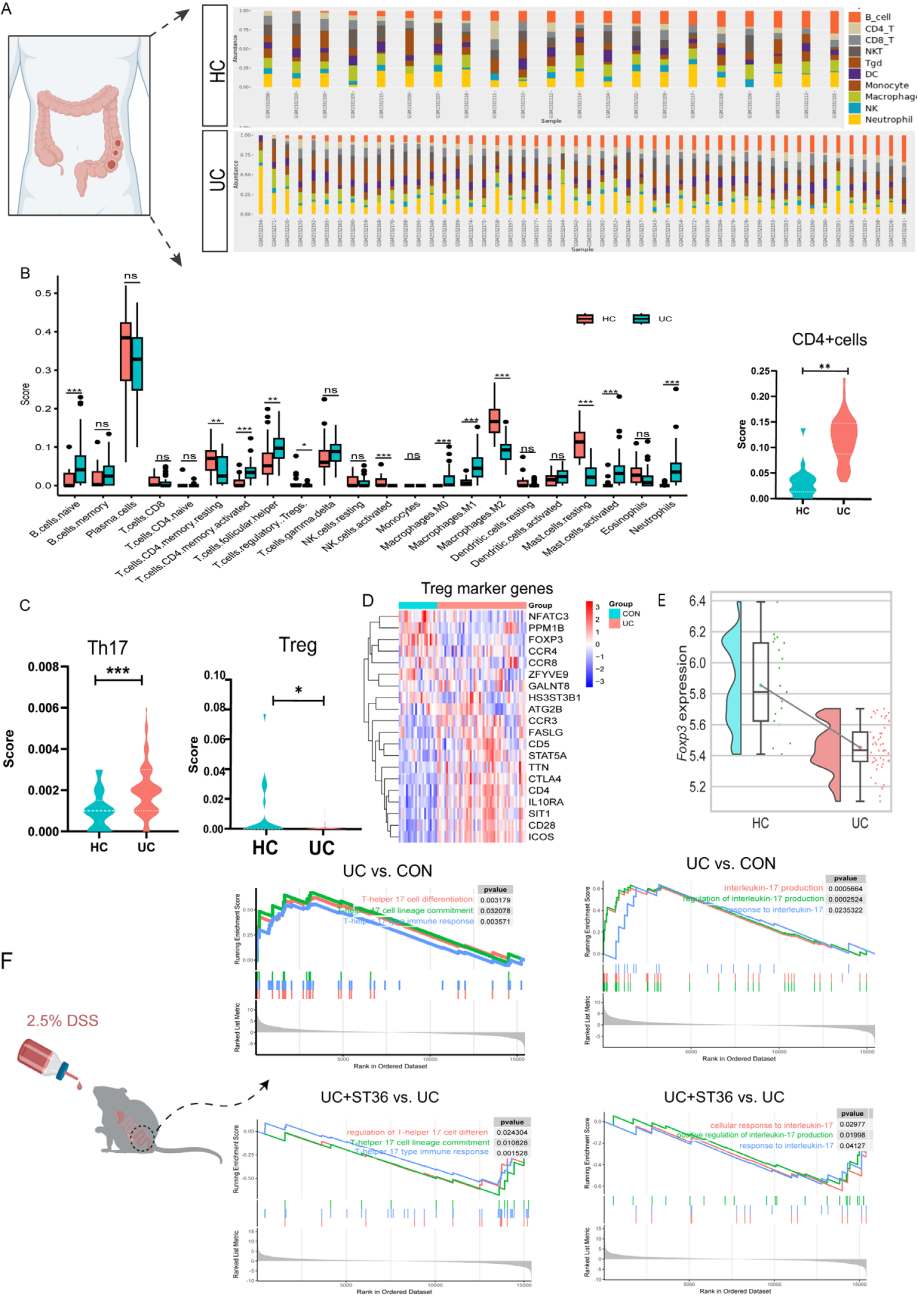
Transcriptomic analysis of Tregs and Th17 cells in UC colon tissues. **(A)** The Percentage Bar Chart of immune cell abundance in UC patient samples. **(B)** The Box Plots of immune infiltration between the UC and healthy groups. **(C)** The Violin Plot Th17 cells and Tregs immune infiltration score in UC and healthy groups. **(D)** Heatmap of Treg marker genes in UC and healthy groups. **(E)** The raincloud plots of Tregs key transcription factor FOXP3 expression in UC and healthy groups. **(F)** Enrichment plots of Th17 cell associated pathways for gene set enrichment analysis(GSEA)HC, health control; UC, ulcerative colitis; Treg, regulatory T cells; TH17, T-helper 17. ns, no significance, *p < 0.05, **p < 0.01, ***p < 0.001 vs. DSS group. HC, healthy control.

Inflammation is hallmark of UC. To delineate the inflammatory cellular profile within the colon of UC patients, we performed an extensive transcriptomic analysis on both human UC patients and UC mouse models. Our analysis revealed an enhanced infiltration of activated CD4+ memory T cells, B naive cells, T follicular helper cells, and macrophages (M0 and M1), as well as activated mast cells and neutrophils, within the colonic tissues of UC patients compared to healthy controls. In contrast, there was a notable decrease in the presence of resting CD4+ memory T cells, regulatory T cells, activated natural killer cells, macrophages (M2) and resting mast cells (**Figures 1A, B**).

Evidences have implicated a disrupted Treg/Th17 axis in UC pathogenesis. Treg and Th17 cells, distinct subsets of CD4+ T cells, are critical for maintaining immune homeostasis and regulating inflammatory responses. In UC patients, our analysis of CD4+ T cells, Tregs, and Th17 cells within immune infiltrates revealed a significant increase in the CD4+ T cell immune score, a decrease in the Treg immune score, and an elevation of the Th17 cell immune score compared with healthy controls (**Figures 1B, C**). Utilizing the ImmuCellAI database, we examined the expression patterns of Treg marker genes. Our analysis revealed down regulation of key Treg markers, such as NFATC3 and PPM1, contrasted with upregulation of *CCR3, FASLG, CD5, STAT5A, TTA, CTLA4, CD4, IL10RA, SIT1, CD28*, and *ICOS* in UC patients. *FOXP3*, the core transcription factor for Treg differentiation, was markedly downregulated. (**Figures 1D, E**).

The balance between Treg and Th17 cells is crucial for immune system stability, and its disruption can result in immune dysregulation, potentially leading to inflammatory diseases or autoimmune disorders. We investigated whether the transcriptional profile of Th17 cells in the colon of UC mice recapitulates the observed changes. Our analysis in UC model mice uncovered a pronounced upregulation of gene sets associated with Th17 cell differentiation and Th17-mediated immune responses. Th17 cells, known for their role in the inflammatory cascade of autoimmune diseases and as primary producers of IL-17 (including IL-17A and IL-17F), displayed a significant upregulation of gene sets implicated in IL-17 production. Intriguingly, our results also demonstrated that moxibustion at acupoint ST36 resulted in the downregulation of these gene sets (**Figure 1F**), implying a potential therapeutic strategy for inhabited Th17 cell differentiation and IL-17 production in the UC mouse colon.

### The expressions of FOXP3 and RORγt in Treg and Th17 cells of the UC mice colon are disrupted

**Figure 2:**
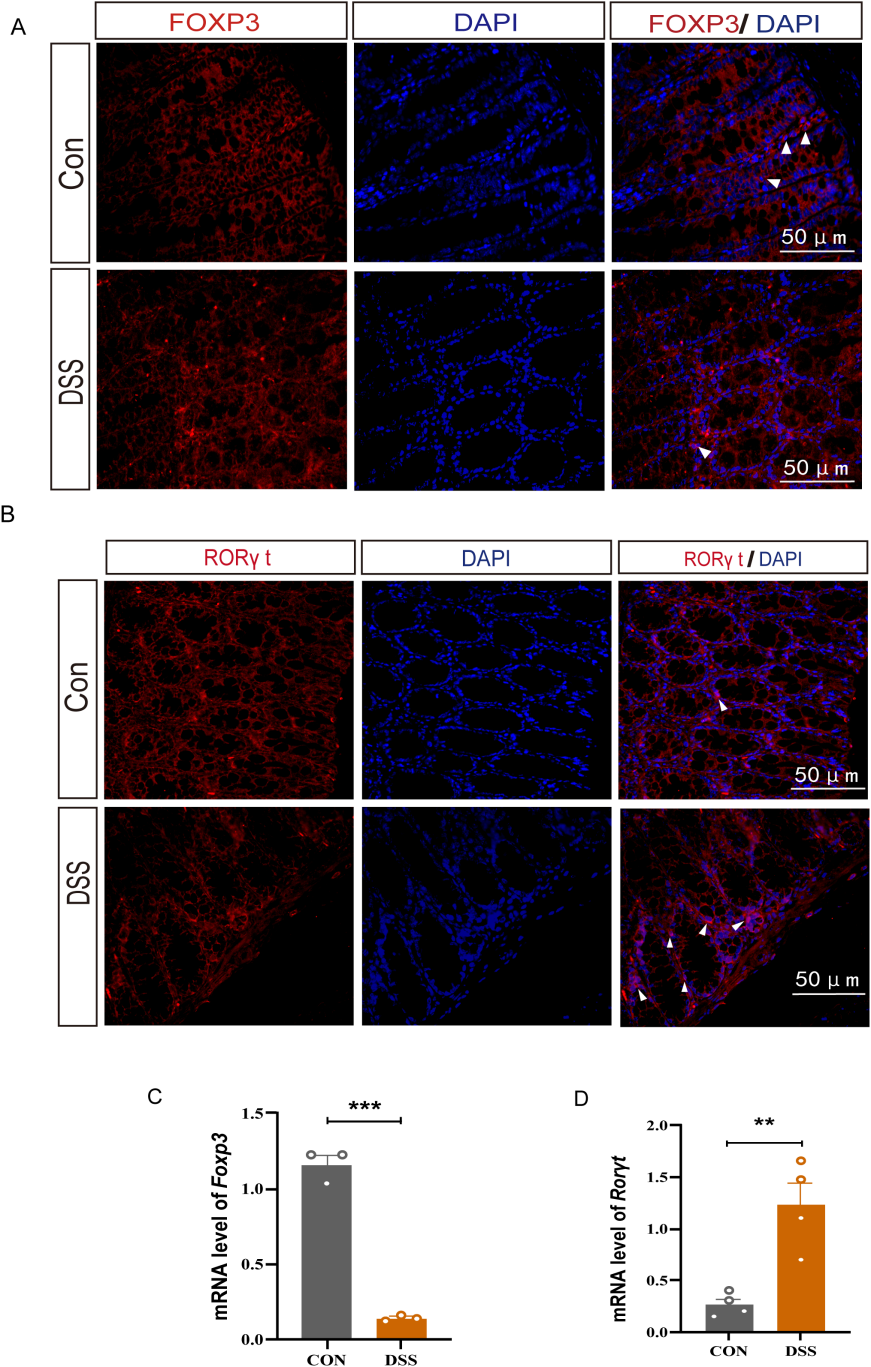
The protein and mRNA expression levels of FOXP3 and RORγt in DSS-induced UC mice. **(A)** Immunofluorescence staining for FOXP3 (white arrow) in colonic tissue (400×, scale bars = 50 μm, n=4). **(B)** Immunofluorescence staining for RORγt (white arrow) in colonic tissue(40×, scale bars = 50 μm, n=4). **(C, D)** Relative mRNA expression of *Foxp3* and *Rorγt* in colon tissue(n=3). Error bars = mean ± SD. **p < 0.01, ***p < 0.001.

FOXP3 and RORγt are essential transcription factors for Tregs and Th17 cells respectively. To confirmed our transcriptomic findings, we assessed the expression of FOXP3 and RORγt in the colonic tissues of DSS-induced UC mice model. Immunofluorescence studies revealed a decrease in FOXP3 expression and an increase in RORγt expression in the UC group. Similarly, the mRNA expression results reflected these changes, with an increased in *Rorγt* and a decline in *Foxp3* mRNA levels in the colonic tissues of UC mice (**Figure 2**). These observations imply that aberrant transcription factor expression or functional disturbed in the UC colon may disrupt the Treg and Th17 cell-mediated immune balance, thereby exacerbating the immunopathological process.

### SIRT1 is activated in LPS-induced inflammation and modulates the Treg/Th17 immune balance axis

**Figure 3:**
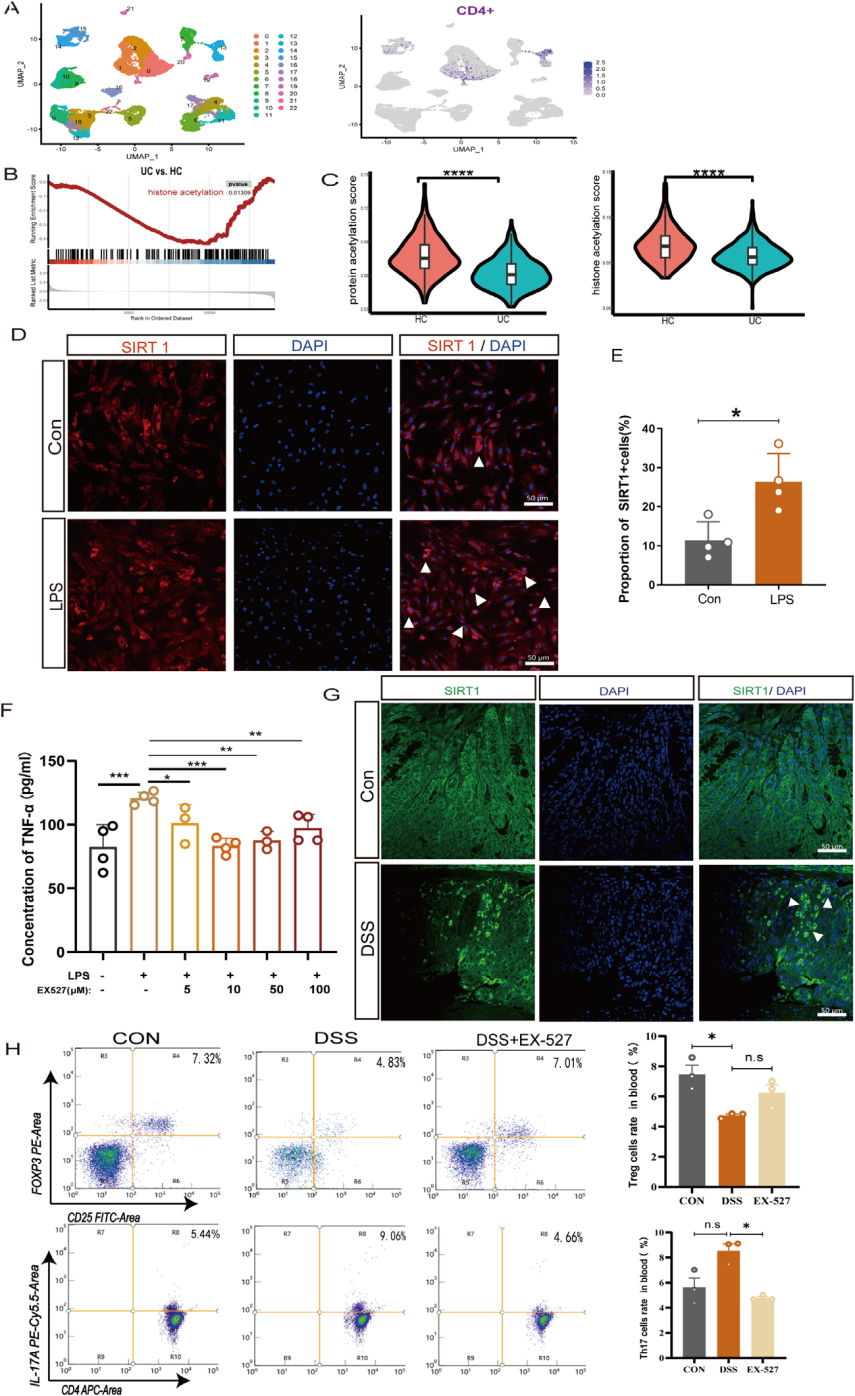
SIRT1 regulates the proportion of Th17 and Treg cells. **(A)** UMAP plots of clusters and the expression level of CD4 + cells in cluster 0 and cluster 13. **(B)** Enrichment plots of acetylation from GSEA in UC colon. **(C)** The pathway activity score of acetylation in colon tissue. **(D, E)** Immunofluorescence staining of SIRT1(white arrow) in LPS-induced colonic epithelial cell inflammation (400×, scale bars = 50 μm, n=4). Error bars = mean ± SD. **(F)** The expression level of TNF-α after exposure to LPS (50 μg/mL) and different concentrations of SIRT1 inhibitor (EX-527) (0–100 μM) for 24 hours (n = 4). Error bars = mean ± SD. **(G)** Immunofluorescence staining of SIRT1 (white arrow) in UC mice colon (400×, scale bars = 50 μm, n=4). **(H)** The percentage of CD25+FOXP3+T cells and CD4+IL-17A +T cells in peripheral blood after EX-527 intervention (n=3). Error bars = mean ± SD. *p< 0.05, **p < 0.01, ***p < 0.001, ****p < 0.0001 *vs.* DSS group. DSS, dextran sulfate sodium salt; TNF-α, tumor necrosis factor; LPS, lipopolysaccharide; IL-10, interleukin 10; TGF-β1, transforming growth factor-β.

Studies have demonstrated that abnormal levels of acetylation modification may play a crucial role in the pathological process of UC (16, 17). To further elucidate the transcriptional changes in UC colon, transcriptome data from UC patients were selected. Through integration, dimension reduction, and clustering analysis of the UC transcriptome data, we identified a total of 22 distinct clusters, by using CD4 molecules as markers, we successfully annotated CD4+ cells and found high expression levels in cluster 0 and cluster 13 (**Figure 3A**). Further investigation into the gene sets within these clusters revealed significant alterations, particularly a marked reduction in genes associated with protein acetylation modification in the colonic tissue of UC patients (**Figures 3B, C**). Evidence once reported post-translational modifications of acetylation is crucial in Th17 cells mediated inflammation (8). Histone acetylation modification is mediated by histone acetyltransferases (HATs) and histone deacetylases (HDACs). By adding or removing acetyl groups to histones, they can regulate protein stability, enzymatic activity, and gene transcription, either directly or indirectly (18).

SIRT1 is an NAD^+^ dependent histone deacetylases involving in acetylation modification, which has been reported to participate in the progression of inflammation (19, 20). To investigate the role of SIRT1 in colonic inflammation, we isolated colonic epithelial cells for further *in vitro* experiment. The results show that in LPS-induced colonic epithelial inflammation, the expression of SIRT1-positive cells significantly increased (**Figures 3D, E**). To further clarify the influence of SIRT1 expression on inflammation, we co-cultured colonic epithelial cells with different concentrations of the SIRT1 inhibitor EX-527, the results showed that after inducing with LPS, the expression of TNF-α exhibited a significant decrease at different concentrations of SIRT1 inhibitor EX-527 (0 μM - 100 μM). Especially, at the concentration of 10 μM, the expression level of TNF-α was the lowest (**Figure 3F**).

Evidences of the imbalance of the Treg/Th17 immune axis contribute to the development of UC, meanwhile, SIRT1 also play a crucial role in regulating of T cell metabolism and functions (21), however, whether SIRT1 enables to affect the Treg/Th17 immune axis remain unknown. Despite their different functions, Th17 cells and pTreg cells arise from a common precursor cell, the naiveCD4 +T cells. Thus, in our experiments, by using naiveCD4+ T cells, we observed that at a concentration of 10 μM, the SIRT1 inhibitor EX-527, promoting the percentage of Treg and decrease the percentage of Th17 cells (**Supplementary Figures S1A, B**). The expression of the immunosuppressive cytokine TGF-β1 and IL-10 significantly increased. (**Supplementary Figure S1C**). Conversely, treatment with the SIRT1 activator SRT1720 revealed that SIRT1 activation decreased the proportion of Treg cells and increased the proportion of Th17 cells (**Supplementary Figures S1D, E**), which corresponded with a reduction in the expression of IL-10 and TGF-β1 (**Supplementary Figure S1F**). These findings indicate that upregulation of SIRT1 promotes inflammatory progression, whereas EX-527 can downregulate SIRT1 expression to mitigate the development of colitis.

*In vivo*, we also observed an increase in the expression of SIRT1 in the colonic tissue of UC mice (**Figure 3H**). To investigate the role of SIRT1 in colitis development, specifically its impact on the Treg/Th17 balance, we utilized EX-527 in subsequent *in vivo* studies. EX-527 was given at a dose of 10 mg/kg, based on the reported in previous studies (22, 23). In peripheral blood, we found the proportion of CD25^+^Foxp3^+^ T cells in the UC mice increased after inhibitor intervention, upon examination of CD4^+^IL-17A^+^ T cells in UC mice showed an increasing trend but without statistical significance (**Figure 3G**). In spleen, after inhibitor intervention, the proportions of CD4^+^IL-17A^+^ T cells in the UC mice decreased (**Supplementary Figure S1J**). Furthermore, we found the given of inhibitor lead to an increase expression of *Foxp3 mRNA* while decreased the expression of *Rorγt mRNA* expression in the UC colon tissues. These results strongly indicate that SIRT1 is closely related to the expression and activity of *Foxp3*+Treg and *Rorγt* +Th17 cells.

### Moxibustion alleviated the symptoms of DSS-induced colitis in mice

**Figure 4:**
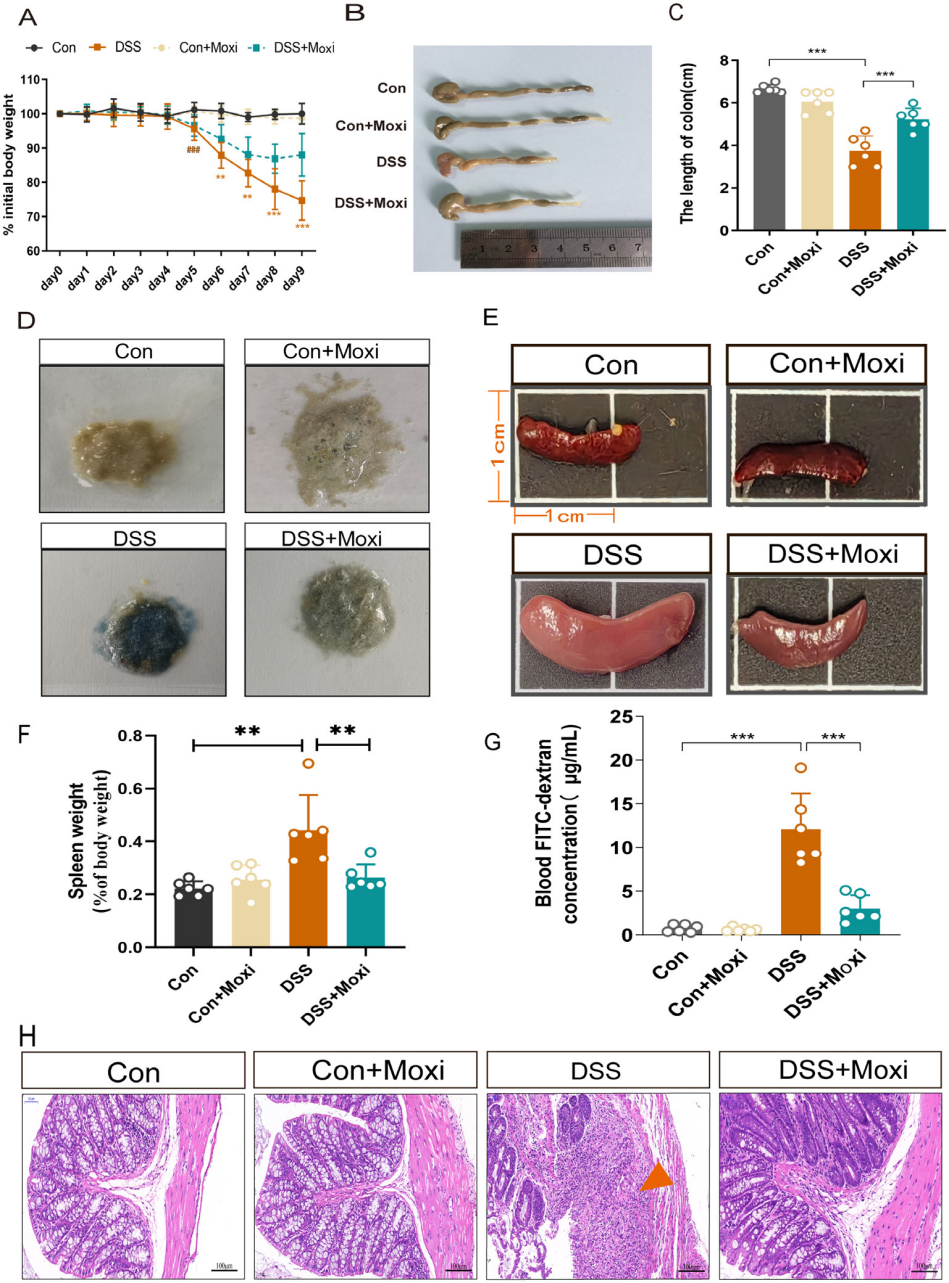
Moxibustion alleviates colonic pathological damage in UC mice. **(A)** Changes in the daily initial body weight loss of mice in each group (n = 8). Error bars = mean ± SD. **(B, C)** Colonic length in different groups (n = 6). Error bars = mean ± SD. **(D)** fecal occult blood test in different groups (n = 6). **(E, F)** The changes in spleen volume and weight in mice. Error bars = mean ± SD. **(G)** Blood FITC-dextran concentration (n = 6). Error bars = mean ± SD. **(H)** Representative images of HE staining of the colon (200 x, scale bars = 100 μM, green arrow represent ulcerative site of colon). ^###^ *p* < 0.001 *vs.* control group; **p < 0.01, ***p < 0.001 *vs.* DSS+Moxi group. FITC, fluorescein isothiocyanate; Con, control; Moxi, moxibustion. HE, hematoxylin-eosin.

Moxibustion, a Traditional Chinese Medicine treatment which was used in preclinical experiments and clinical trials, has shown significant efficacy in alleviating UC symptoms such as abdominal pain and diarrhea. To evaluate the therapeutic effect of moxibustion on UC, we assessed the body weight loss of UC mice, the results displayed DSS-induced UC mice exhibited significant weight loss on the 5th day, notably, after moxibustion treatment, the weight loss in UC mice was reduced (**Figure 4A**). Macroscopic evaluations of colonic inflammation included measuring the length of the colon and the weight of the spleen. In the UC mice, a decrease in colon length was observed (**Figures 4B, C**), while the spleen weight increased almost double times when compared to the Control group (**Figures 4E, F**). The hematochezia is a main symptom of UC diseases, thus we assessed the fecal occult blood testing (FOBT) in mice, the results showed moxibustion treatment significantly reduces the bleeding in UC mice (**Figure 4D**). The disruption of the intestinal barrier is commonly observed in UC (24). Therefore, we detected the intestinal mucosal permeability by testing blood FITC-dextran, the results revealed the intestinal permeability in UC mice significantly increased while moxibustion decreased the intestinal permeability in UC mice (**Figure 4G**). Meanwhile, the results of HE staining showed that in UC mice, the structure of colonic tissue disrupted, characterized by significant ulcerative sites, structural disorder, and of inflammatory cells. However, after moxibustion treatment, there was partial recovery in colon morphology, relief of disruption in mucosal and glandular structure, absence of noticeable ulcerative sites. (**Figure 4H**). These results shows that moxibustion can alleviate intestinal inflammation and promote the recovery of intestinal mucosal barrier function in UC mice.

### Inhibition of SIRT1 facilitated moxibustion to restore the balance of Treg/Th17

**Figure 5:**
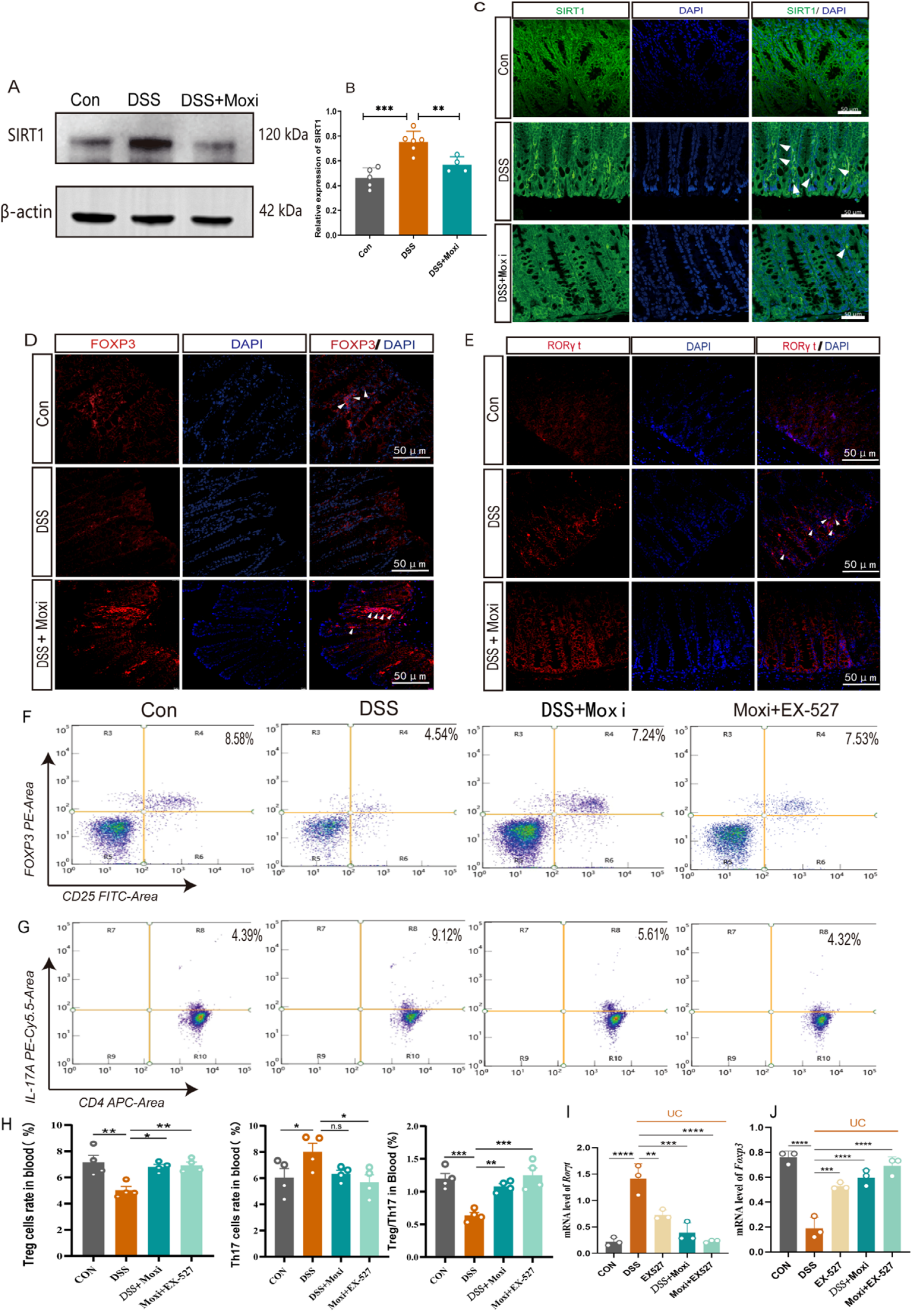
Moxibustion downregulated the expression of SIRT1 in the colon and restored the ratio of Treg to Th17 cells. **(A, B)** Quantification of the western blot results of SIRT1 in colon tissue (n = 4-6). Error bars = mean ± SD. **(C)** Immunofluorescence staining of SIRT1 (white arrow) in colon tissue (400×, scale bars = 50 μM, n = 3). **(D, E)** Immunofluorescence staining for FOXP3/RORγt (white arrow) in colonic tissue (400×, scale bars = 50 μM, n = 4). **(F-H)** The percentage of CD25+FOXP3+ T cells and CD4+IL-17A + T cells in peripheral blood of after moxibustion and EX-527 intervention(n=4). Error bars = mean ± SD. **(I, J)** Relative mRNA expression of *Foxp3* and *Rorγt* in colon tissue after moxibustion and EX-527 intervention (n=3). Error bars = mean ± SD. ns, no significance, *p < 0.05, **p < 0.01, ***p < 0.001, ****p < 0.0001, compared with DSS group. FOXP3, forkhead/winged helix transcription factor; RORγt, retinoid related orphan receptor gamma t; T cell, T lymphocyte cell.

Post-translational modifications (PTMs) of acetylation is crucial in Treg/Th17 axis mediated inflammation response (8). Previous studies demonstrated moxibustion is able to modulate the expression of STAT3, HIF-1α, RORγt and FOXP3 in UC mice, which contribute to the rebalancing of Th17/Treg axis (25, 26). To elucidate whether moxibustion affects the expression of FOXP3 and RORγt through modulation of PTMs and thereby regulates the balance of the Treg/Th17 axis, we proceeded with the following experiments. By utilizing pan-antibody assays, we first investigated the expression profiles of various PTMs, including acetylation, phosphorylation, ubiquitination, succinylation, crotonylation within the colonic tissues of UC mice, as well as the potential modulatory effects of moxibustion on these modifications. The results revealed that moxibustion exerted a regulatory effect on acetylation, predominantly characterized by a decrease in acetylation levels within the colonic tissues of UC mice, which was subsequently elevated after moxibustion treatment. This led us to hypothesize whether SIRT1 related pathways can promote or inhibit the role of moxibustion. We firstly assessed the changes of SIRT1 expression in the colonic tissue after moxibustion treatment. In UC mice, the expression of SIRT1 was increased, however, after moxibustion intervention, SIRT1 expression was found to be decreased (**Figures 5A, B**). Additionally, we measured the expression levels of FOXP3 and RORγt in colonic tissues. Immunofluorescence results indicated that compared to control group, FOXP3 expression decreased while RORγt expression increased. Notably, moxibustion treatment enhanced FOXP3 expression while reducing RORγt levels in the colons of UC mice (**Figures 5D, E**). These results provide compelling evidence that moxibustion treatment capable of regulating the expression of SIRT1, FOXP3 and RORγt.

To further clarify whether moxibustion can drive SIRT1 and subsequently impact the Th17/Treg axis, we examined the effects of moxibustion on Th17 and Treg cell proportions following SIRT1 inhibition. The expression of *Foxp3* and *Rorγt* respectively reflect the expression of Treg cells and Th17 cells in the body. Our results showed the expression of *Rorγt* mRNA in the colonic tissues increased while the expression of *Foxp3* mRNA decreased. However, after receiving moxibustion or inhibitor intervention, the expression of *Rorγt mRNA* decreased, and the expression of *Foxp3 mRNA* increased, the combined use of moxibustion and inhibitors can more effectively regulate the expression of *Foxp3* and *Rorγt mRNA* (**Figures 5I, J**). In peripheral blood, we found the proportion of CD25^+^Foxp3^+^ T cells in the UC mice increased significantly after moxibustion intervention. Notably, the combined application of moxibustion and inhibitors resulted in a more pronounced increase in the proportion of Treg cells. Although there was a trend towards a decrease in CD4^+^IL-17A^+^ T cells following moxibustion, this change was not statistically significant. In contrast, the combined use of moxibustion and inhibitors resulted in a marked reduction in CD4^+^IL-17A^+^ T cells (**Figures 5F–H**). In spleen, it was also found that the proportion of CD4^+^IL-17A^+^ T cells in UC mice increased, while the inhibitor enforced the ability of moxibustion to decreased the proportion of CD4^+^IL-17A^+^ T cells. (**Supplementary Figure S3**). These results indicate that the expression of SIRT1 can be modulated by moxibustion, and in the context of SIRT1 inhibition, moxibustion significantly enhances the regulation of the Th17/Treg balance.

### SIRT1 inhibitors enhance the therapeutic effects of moxibustion on intestinal barrier protection

**Figure 6:**
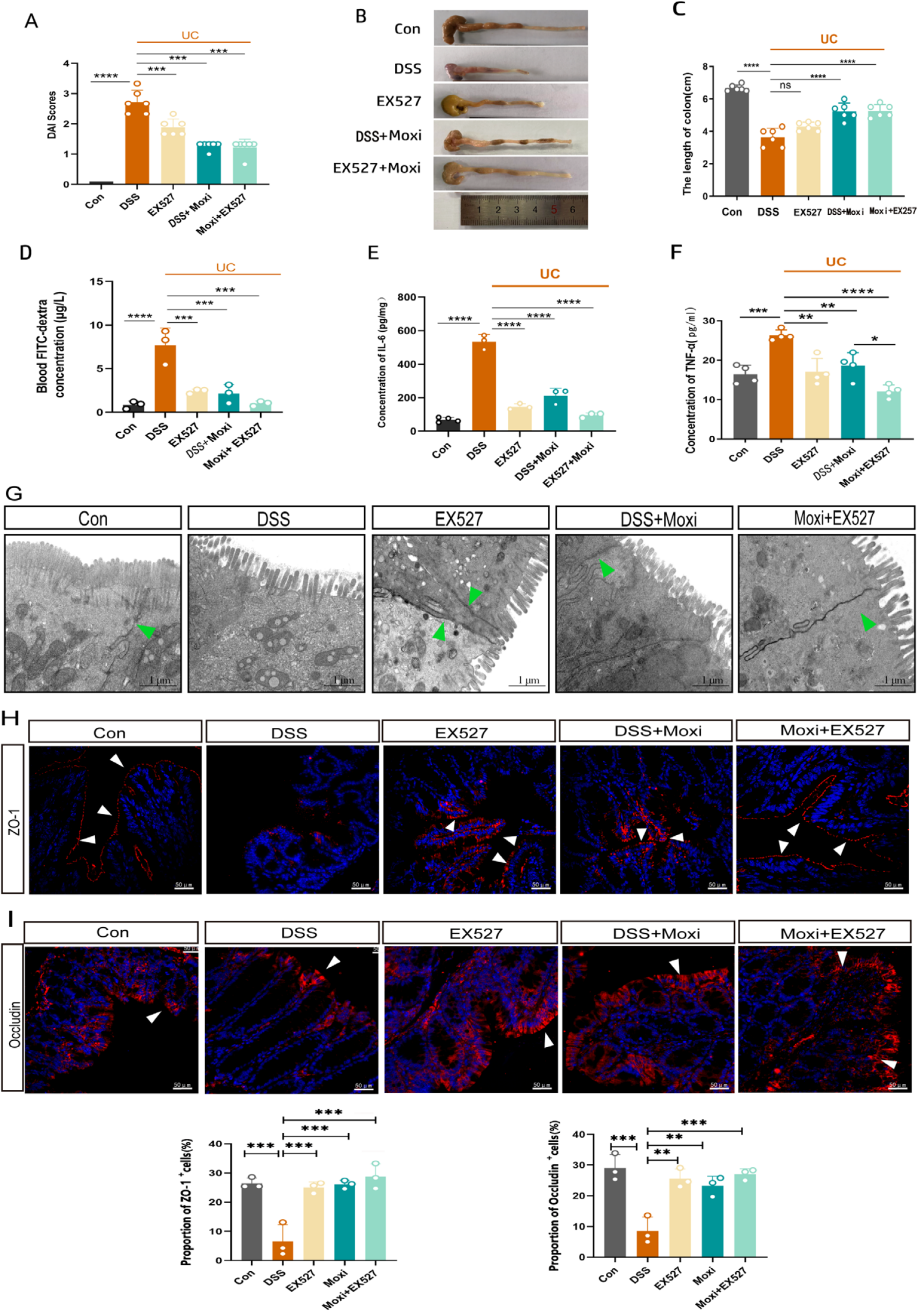
EX-527 enhances the protective effects of moxibustion on the intestinal barrier. **(A)** DAI score of UC mice after (n=6). Error bars = mean ± SD. **(B, C)** The length of colon after moxibustion and EX-527 intervention (n = 6). Error bars = mean ± SD. **(D)** Blood FITC-dextran concentration after moxibustion and EX-527 intervention (n = 3). Error bars = mean ± SD. **(E, F)** Concentration of IL-6 and TNF-α in different group (n = 3). **(G)** Electron microscopy images of cell tight junction (green arrows) in different group (scale bar= 1μM, 10000×, n=3). **(H, I)** Immunofluorescence staining of ZO-1 and Occludin (white arrow) in colon tissue after EX-527 and moxibustion intervention (scale bar=50μM, 400×, n=3). Error bars = mean ± SD. *p < 0.05, **p < 0.01, ***p < 0.001, ****p < 0.0001, compared with the DSS group. ZO-1, zonula occludens-1; DAI, disease activity index.

To further investigated the contribution of the moxibustion to the observed responses mediated by SIRT1 inhibition, the inhibitors EX-527 was administered *in vivo* and the mice showed a reduction in the DAI (P < 0.001) and an increase in the colon length (P < 0.01) (**Figures 6A–C**). Meanwhile, we also found that UC mice exhibited a higher expression of TNF-α and IL-6, after moxibustion and EX-527 intervention, the levels of TNF-α and IL-6 significantly decreased (**Figures 6E, F**). The integrity of intestinal barrier is important for intestinal defense. The tight junctions (TJs) which including ZO-1 and Occludin both are important components of intestinal barrier. To identify the roles of SIRT1 in the pathogenesis of intestinal barrier, we observed the role of SIRT1 in TJs regulations. Electron microscopy results revealed that mice with UC exhibited disrupted intercellular junctional structures, mitochondrial swelling, and vacuolization, however, following the intervention of moxibustion and EX-527, the integrity of tight junctions (TJs) was restored, indicating a positive effect on the repair of intercellular connections and the improvement of mitochondria homeostasis (**Figure 6G**). Moreover, results demonstrated that the combination of moxibustion and EX-527 showed the most substantial reduction in plasma FITC-dextran content (**Figure 6D**). Additionally, we observed a decrease of ZO-1 and Occludin in the mucosal layer in UC mice, whereas moxibustion and EX-527 significantly increased their expression in the intestinal mucosal layer (**Figures 6H, I**). These findings provide evidences that EX-527 intervention enhances the protective effects of moxibustion on the integrity and function of the intestinal barrier in mice with UC.

## Discussion

**Figure 7:**
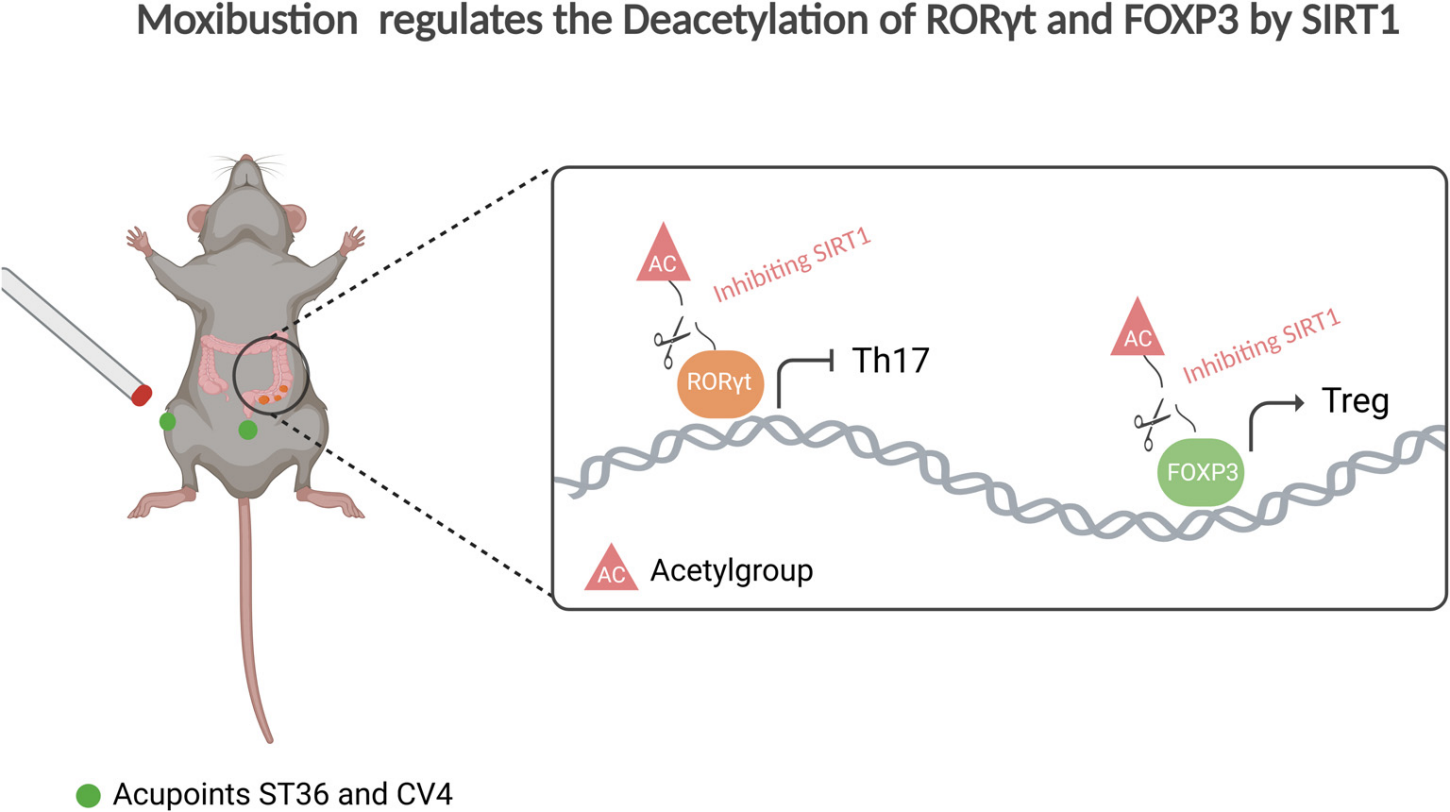
Moxibustion regulates the deacetylation of RORγt and FOXP3 by SIRT1. Moxibustion at the ST36 and CV4 acupoints in UC mice can suppress the expression of SIRT1 in mouse colonic tissue, consequently restoring the Th17/Treg axis and mitigating inflammation by influencing the expression of the upstream core transcription factors RORγt and FOXP3 in Th17/Treg cells respectively.

In this study, we report a significant reduction in protein acetylation levels in the colonic tissues of patients with UC, accompanied by an overactivation of Th17-mediated immune responses in the UC context. This alteration is likely linked to an abnormal of acetylation modifications. Through detailed investigations, we demonstrate that SIRT1, a key deacetylase, mediating the acetylation levels of the upstream core transcription factors RORγt and FOXP3 in Treg and Th17 cells, thereby influencing the differentiation of naive T cells into Th17 cells or Tregs (**Figure 7**). Notably, the inhibition of SIRT1 expression promotes Treg differentiation and enhances the secretion of anti-inflammatory cytokines, while concurrently suppressing Th17 differentiation and the release of pro-inflammatory cytokines.

An imbalance between Tregs and Th17 is implicated in the pathophysiology of UC. In the active phase of UC, particularly in the context of Epstein–Barr virus infection, there is a marked decrease in the expression of Foxp3 in Tregs, which inversely correlates with serum C-reactive protein and Mayo score, suggesting their potential role in UC remission (27, 28), Tregs and Th17 cells, primarily found in the spleen and peripheral blood, are mobilized and transferred to intestinal ulcer sites at UC onset, particularly in Peyer’s patches and the spleen, leading to disrupted homeostasis of Treg and Th17 cells and further promoting inflammation and intestinal damage (29, 30).

Treg and Th17 cells both differentiate from CD4^+^T cells. The differentiation of Tregs and Th17 cells within distinct cytokine environments is crucial in the pathogenesis of conditions such as gastrointestinal and pulmonary disorders, as well as neurological diseases (31–34). FOXP3 and RORγt are essential transcription factors for Tregs and Th17 cells, respectively. The equilibrium between these factors is essential for the differentiation of naive T cells and for maintaining immune homeostasis across organs, including the gastrointestinal.

PTMs are crucial for regulating intracellular proteins and enabling rapid cellular responses to external signals. With over 600 types identified, acetylation stands out as a highly conserved modification affecting various biological processes, including transcription, signaling, protein stability, metabolism, and pathogen responses (35, 36).

Acetylation is a fundamental regulatory mechanism for both FOXP3 and RORγt, influencing T cell subset differentiation. Evidence indicates histone deacetylase (HDAC) can suppress RORγt transcription and the expression of RORγt-dependent genes. *In vitro* studies have shown that Th17 cells can be induced to express RORγt upon treatment with HDAC inhibitors such as Sodium butyrate and Apicidin (37). Conversely, applying these inhibitors during the differentiation of naive CD4 cells into Th17 cells results in reduced *RORγt* gene expression, suggesting that deacetylase inhibitors play a role in modulating *RORγt* expression at specific stages of T cell differentiation (8).

SIRT1, a member of the sirtuin family and a highly conserved NAD^+^ dependent deacetylase, acts as a post-translational regulator that mediated various biological processes, including cell aging and apoptosis, glucose and lipid metabolism, oxidative stress as well as involved in modulating inflammation (9, 38). In intestinal, SIRT1 can promote the onset and progression of inflammatory bowel disease(IBD), loss of *Sirt1* can increase the number of Paneth cells and goblet cells and alleviate colitis (39). Meanwhile, SIRT1 has been found to have a pro-inflammatory effect on the generation and function of Th17 cells by promoting IL-17 production through the stabilization of *RORγt* (40). In our study, we found that SIRT1 is upregulated and the Th17/Treg axis is disrupted in UC mice. Treatment of UC mice with the SIRT1 inhibitor EX-527 resulted in increasing mRNA expression of FOXP3 in colonic tissues and a higher ratio of Treg cells. This may be related to potential links between SIRT1 deacetylation sites on FOXP3, including K31, K262, K267 and K142 (41, 42). Furthermore, the application of SIRT1 inhibitor EX-527 significantly reduced the mRNA expression levels of RORγt in Th17 cells, resulting in a decreased ratio of Th17 cells.

In addition, our research also indicates that moxibustion can effectively regulate the expression of SIRT1 in UC colon tissue. SIRT1 mediated deacetylation of RORγt and FOXP3 also participates in the mechanism of moxibustion. Inhibiting SIRT1 levels will promote the therapeutic effect of moxibustion. Moxibustion has been used since ancient China to treat diarrhea, abdominal pain and improving the quality of life in patients with IBD (43, 44). However, due to the unclear mechanism of moxibustion and the uncertain ideal therapeutic effect, its widespread application is limited. Our results indicate that combination using moxibustion and EX-527 have a positive therapeutic effect on DSS-induced UC mice, including improving the general condition of UC mice, alleviating UC symptoms, restoring lost body weight, increasing colon length, significantly reducing the DAI score and decreasing intestinal mucosal permeability. Furthermore, this joint application improves colonic tissue morphology, maintain the integrity of colonic mucosal barrier, and reduces mucosal edema and inflammatory cell infiltration, thereby providing therapeutic benefits for UC.

This study is a laboratory research and further clinical validation is necessary. In addition, it is unclear whether other deacetylases also play similar roles. Furthermore, SIRT1 has multiple functions, other pathways such as inflammatory pathways, insulin related pathways (45), mitochondrial (46) and oxidative stress (47) may also be involved and should be investigated in the future.

## Conclusion

Our research shows that inhibition SIRT1 can improve UC by regulating FOXP3 and RORγt, which are upstream transcription factors of the Treg and Th17 immune balance axis. This finding indicates a new important potential target for the treatment of UC.

## Data Availability

The datasets analyzed for this study can be found in the Gene Expression Omnibus (GEO) database, GSE227407 and GSE87466.
