# Supplementary material for: Inhibition SIRT1 to regulate FOXP3 or RORγt can restore the balance of Treg/Th17 axis in ulcerative colitis and enhance the anti-inflammatory effect of moxibustion
Source: Front Immunol. 2025 Jan 10;15:1525469. doi: 10.3389/fimmu.2024.1525469 (PMC11757129; doi:10.3389/fimmu.2024.1525469)
Supplement: Supplementary file 1 [file DataSheet1.pdf]

frontiers

The International Society for the Advancement of Cytometry (ISAC) has highlighted the importance of including comprehensive methodological information to ensure data reproducibility and reliability. In line with this, Frontiers in Immunology now requires authors to submit a checklist for manuscripts that involve flow or mass cytometry. This checklist helps standardize the reporting process, improving the quality and transparency of published data. By doing so, we support scientific progress, making it easier for other researchers to replicate and validate experiments.

This form should be submitted with any manuscripts using flow or mass cytometry.

Sample/specimen/material description

☑ Total blood

☑ PBMCs

☑ Organ digests

Other____

Did the samples suffer any treatment before or after incubation with the antibodies?

☑ Drug Selisistat,SRT1720HCL

☐ Cell permeabilization ______________________

☐ Dye ______________________________________

☐ Propidium iodine

☐ Not applicable

Other Moxibustion

Instrument and antibodies

Name of the Cytometer ZE5,BioRad

Antibodies and targets	Fluorochrome/ Metal	Catalog number/Company
e.g. anti-CD4	FITC	Cat. XXX/ XXX ltd.
anti-CD4	APC	Cat.1540-11/Southern Biotech

anti-CD25	Alexa Flour 488	Cat.53-0251-82/Bioscience
anti-IL-17A	PerCP 5.5	Cat.560408/BD
anti-FOXP3	PE	560408/BD

Data analyses

1. Name of the software Everest
2. Reference gating strategy in the manuscript or supplementary material

Gating strategy in (eg Figure X) Figure3(H),Figure5(F-H),Supplementary Figures S1-S2.
