# Supplementary material for: Inhibition SIRT1 to regulate FOXP3 or RORγt can restore the balance of Treg/Th17 axis in ulcerative colitis and enhance the anti-inflammatory effect of moxibustion
Source: Front Immunol. 2025 Jan 10;15:1525469. doi: 10.3389/fimmu.2024.1525469 (PMC11757129; doi:10.3389/fimmu.2024.1525469)
Supplement: Supplementary file 2 [file Image1.pdf]

Supplementary Figures.

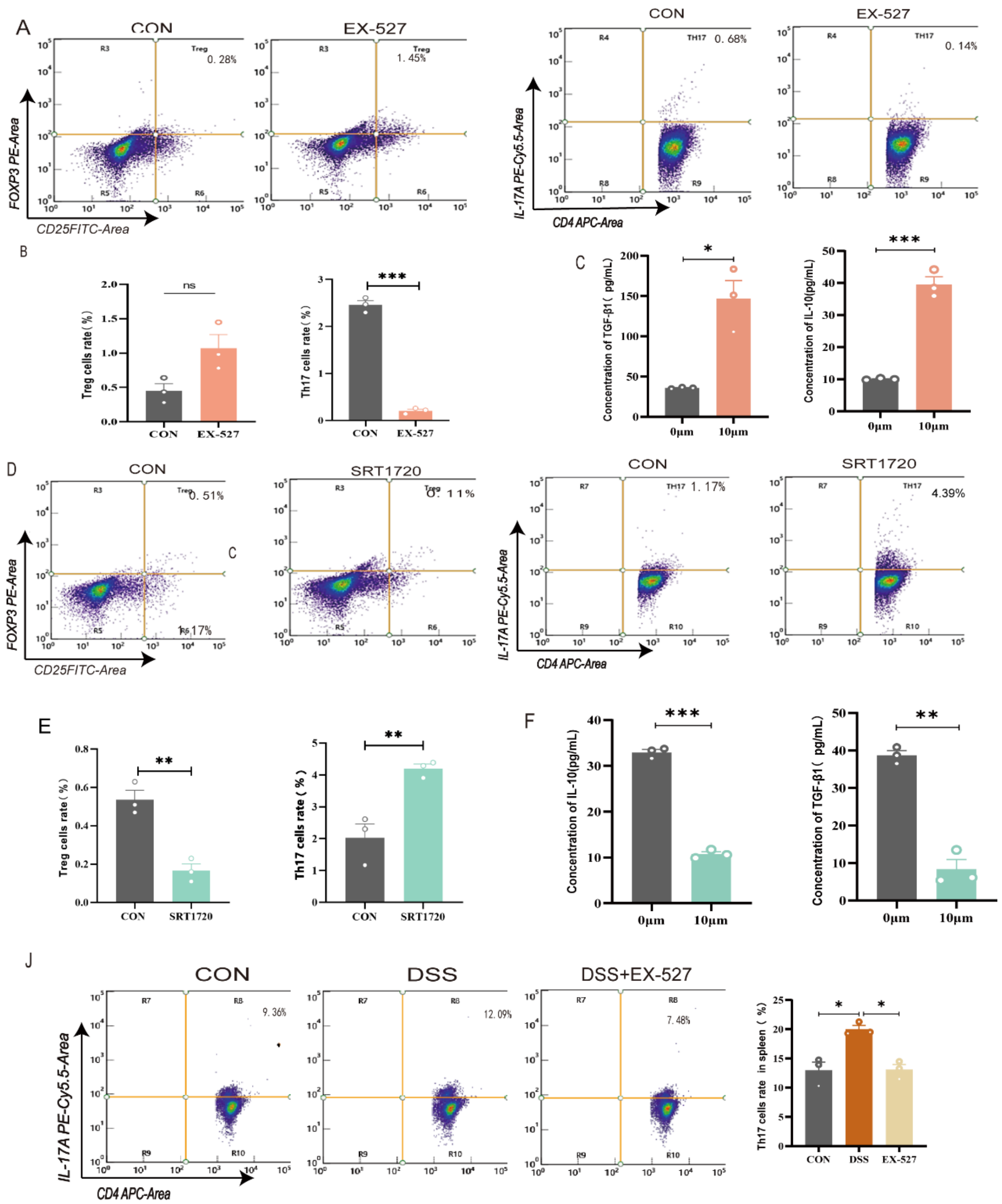

**Figure S1.** (A) The expression level of TNF-α after exposure to LPS (50 μg/mL) and different concentrations of SIRT1 inhibitor (EX527) (0–100 μM) for 24 h (n = 4) (B)The ratio of Treg and Th17 after intervention by EX-527 (C) The expression of IL-10 and TGF-β1 after SIRT1 inhibitor (EX-527) intervention for 72h at 10 μM (n = 3). (D) The ratio of Treg and Th17 after intervention by SIRT1 activator (SRT1720). (E) The ratio of Treg and Th17 after intervention by SRT1720.(F)The expression of IL-10 and TGF-β1 after SRT1720 intervention for 24h at 10 μM (n = 3). (J) The percentage of CD4+IL-17A +T cells in spleen(n=3). Error bars = mean ± SD.

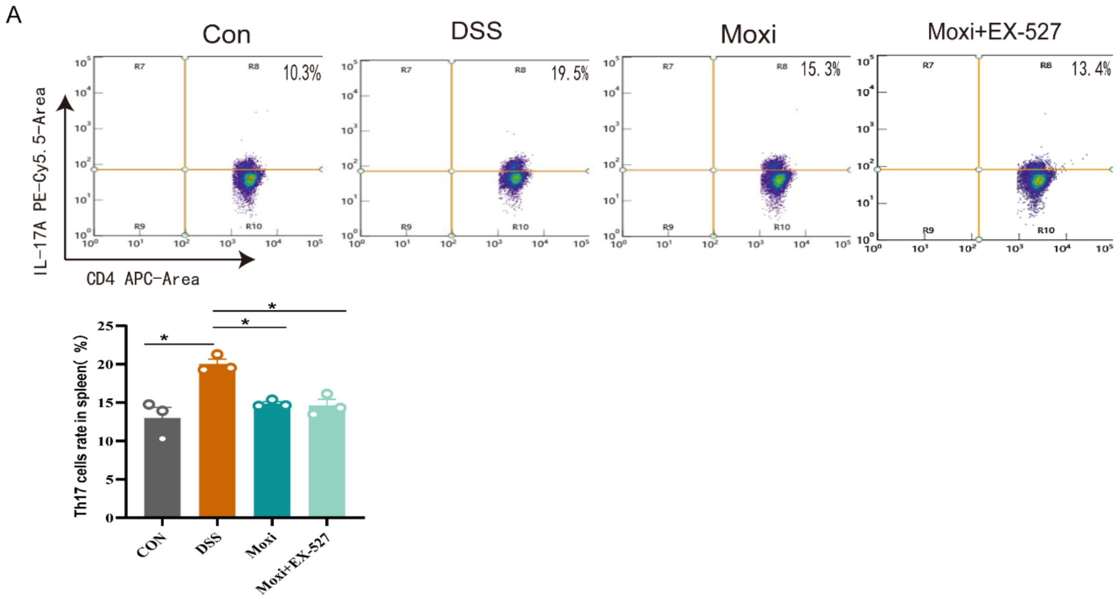


Figure S2. The percentage of CD4+IL-17A +T cells in spleen(n=4). Error bars = mean ± SD.

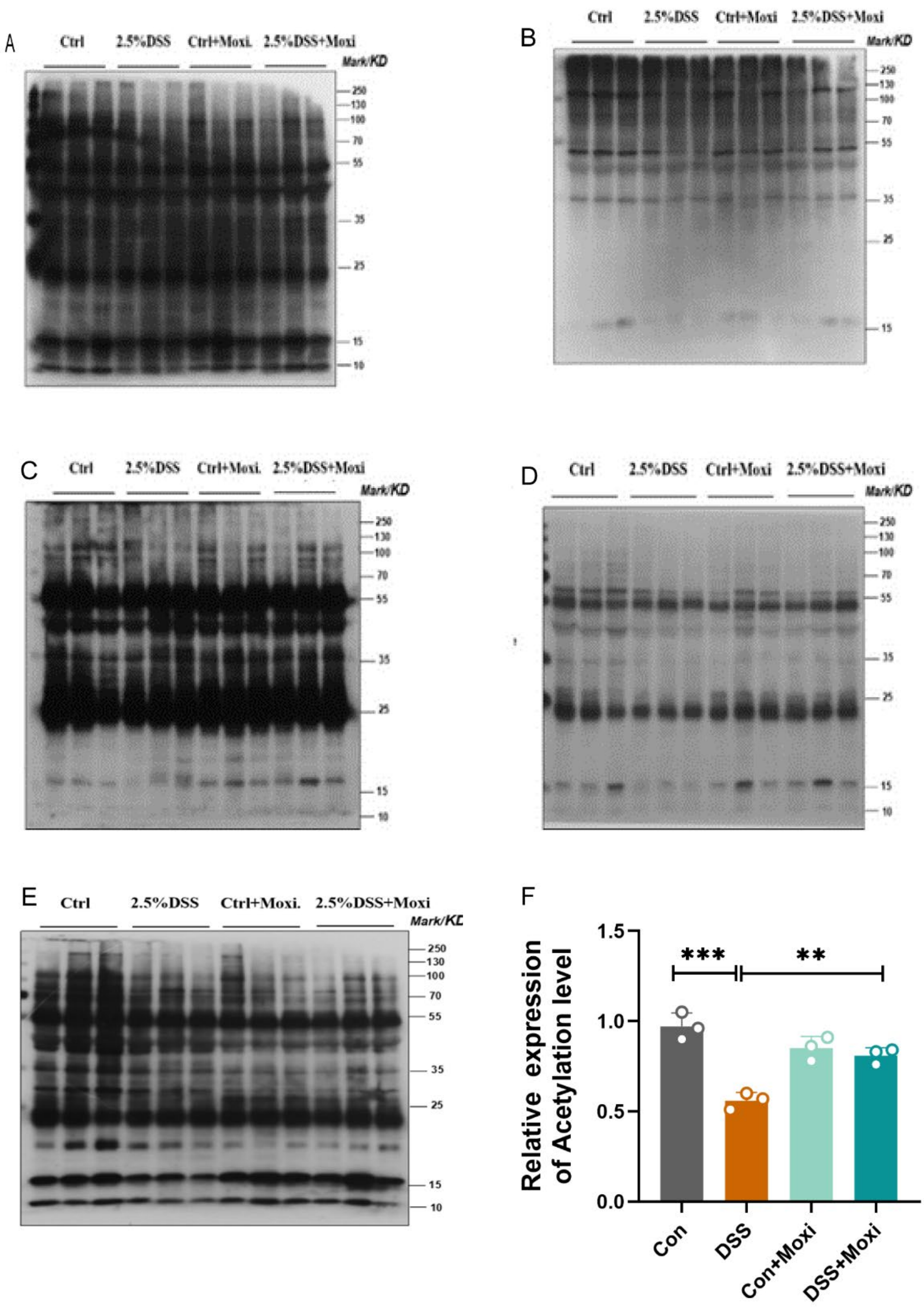


**Figure S3.** Moxibustion upregulated the lysine acetylation level of colonic proteins in UC mice. (A) Results of pan-antibody detection for lysine phosphorylation. (B) Results of pan-antibody detection for lysine ubiquitination. (C) Results of pan-antibody detection for lysine succinylation. (D) Results of pan-antibody detection for lysine crotonylation. (E) Results of pan-antibody detection for lysine acetylation. (F) Relative expression of protein acetylation. Error bars = mean ± SD
