# Supplementary figures and images for: Inhibition SIRT1 to regulate FOXP3 or RORγt can restore the balance of Treg/Th17 axis in ulcerative colitis and enhance the anti-inflammatory effect of moxibustion
Source: Front Immunol. 2025 Jan 10;15:1525469. doi: 10.3389/fimmu.2024.1525469 (PMC11757129; doi:10.3389/fimmu.2024.1525469)

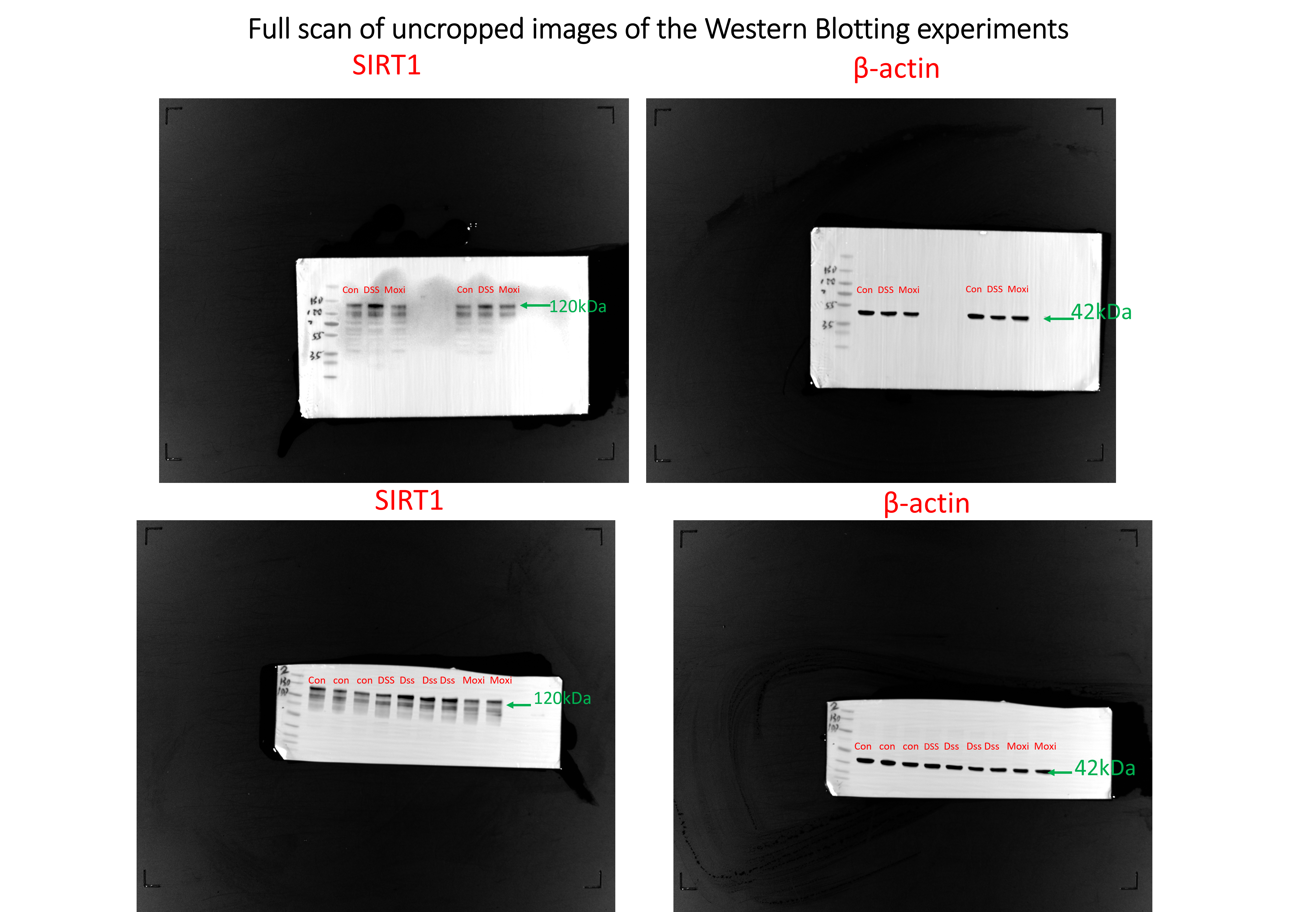

Supplement: Supplementary file 3 [file Image2.tif]

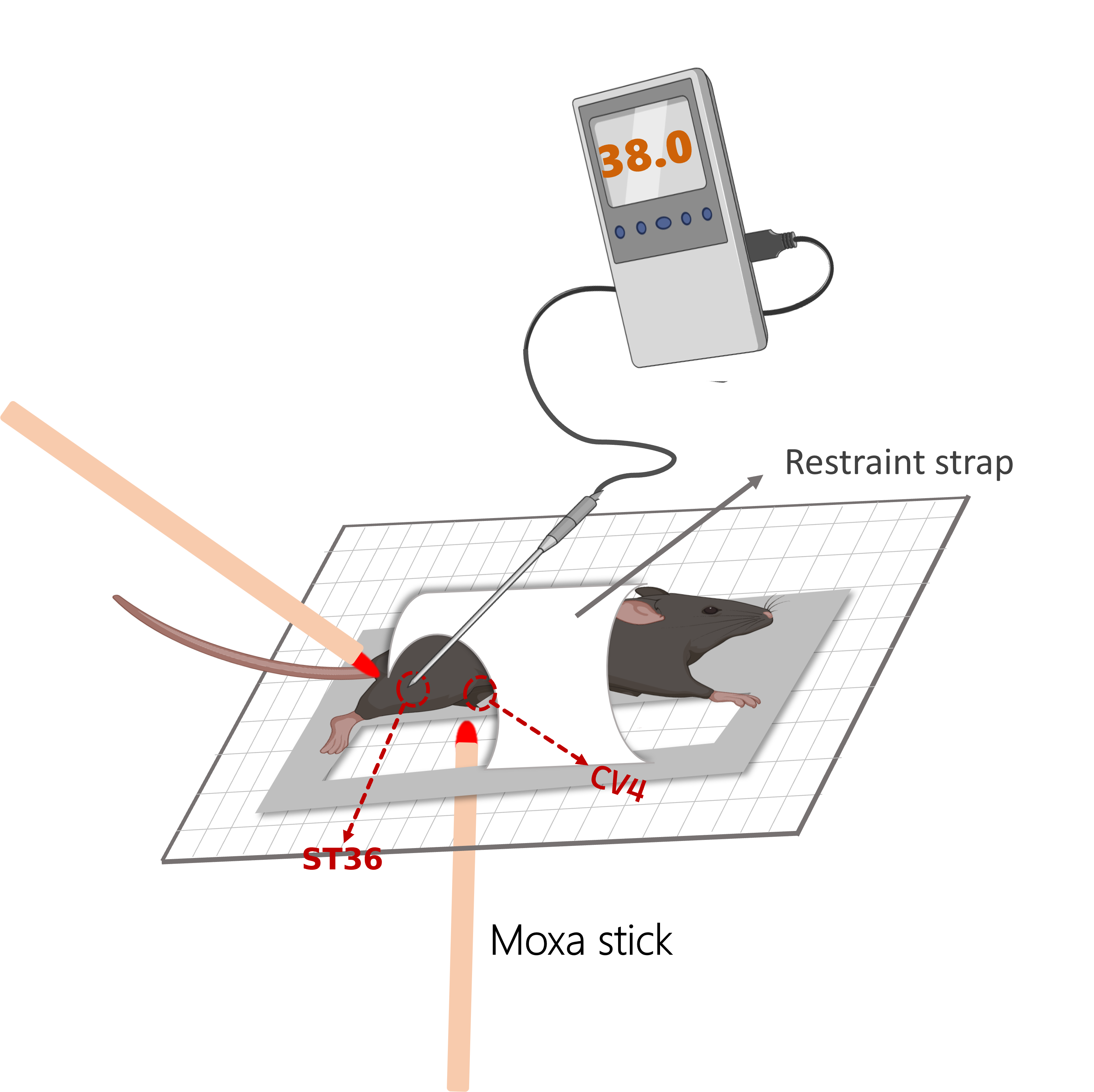

Supplement: Supplementary file 4 [file Image4.tif]
